# Mongolia: Failure of Total Banning of Asbestos

**DOI:** 10.5334/aogh.4035

**Published:** 2023-08-02

**Authors:** Naransukh Damiran, Arthur L. Frank

**Affiliations:** 1 Mongolian National Association of Occupational Hygienists, Ulaanbaatar, Mongolia; 2 Dornsife School of Public Health, Drexel University, Philadelphia, PA 19104, USA

**Keywords:** asbestos exposure, asbestos related disease, asbestos control

## Abstract

The primary uses of asbestos in Mongolia are in thermal power plants, construction and at railway companies. There is, however, limited data on both asbestos consumption and asbestos related disease (ARD) in Mongolia. The purpose of this paper is to report on the failure to completely ban asbestos in Mongolia. To write this paper, available asbestos related literature, published nationally and internationally, and legal regulations, national standards and guidelines on asbestos control were reviewed. Mongolia consumed a total of 44,421.9 metric tons of asbestos containing materials (AMCs) between 1996 and 2014. As a key indicator of ARD, 54 cases of mesothelioma were diagnosed at the National Cancer Center by pathological testing of tissue samples between 1994 and 2013. In 2010, The government made the decision to stop all types of asbestos use under the Law on Toxic and Hazardous Substances. However, there was no nationwide action plan to gradually reduce asbestos use, promote substitutes and raise awareness of health hazards and economic burdens in the future from asbestos use. There was also no planning for safe removal of asbestos currently in place. After the banning of asbestos, thermal power plants told the government that they could not produce electricity without insulation of AMCs and substitution materials were economically not feasible. Due to pressure from the energy sector and inadequate awareness of asbestos hazards, the government changed the legal status on asbestos in 2011 as a restricted chemical. Asbestos is still allowed to be used, and workers and the general community are still unnecessarily exposed to this carcinogen.

## 1. Introduction

Although now banned in about seventy countries, Mongolia still uses asbestos, which is a well known carcinogen [1,2]. The primary uses of asbestos in Mongolia are in thermal power plants, construction and at railway companies [3]. A study showed that since 1961 imported asbestos and asbestos containing materials (ACMs) from Russia was widely used for insulation in power plants, pipelines, building and construction materials when the first coal fired power plant began operating [4]. There is limited data available on asbestos consumption and asbestos related diseases (ARDs) in Mongolia due to a lack of a registration and surveillance system on asbestos consumption on ARDs among high-risk populations [3].

The World Health Organization (WHO) estimated that 125 million people are exposed to asbestos at the workplace and 250,000 people die every year from asbestos related-lung cancer, mesothelioma and asbestosis and other diseases resulting from occupational exposures [5]. The WHO and the International Labor Organization (ILO) outlined Recommendation for National Program for Elimination of Asbestos-Related Diseases for countries to gradually reduce asbestos uses, eventually to stop it and eliminate ARDs [6].

There is no estimated number of workers in Mongolia who are currently exposed to asbestos at workplaces. Workers at coal fired power plants who maintain pipelines or work at repair shops for locomotives, automobiles and heavy equipment, chemical analytical laboratories and construction renovation companies all have potential exposure to asbestos at their workplaces [3,7]. According to the National Statistical Information Service, 248,600 people work in sectors of energy, construction and auto vehicle repair shops which is 7.5% of the total Mongolia population of 3.3 million [8].

Mongolia introduced its first legislation to control toxic and hazardous chemicals in 1995 with the Law on Protection against Toxic Chemicals. But this law did not cover asbestos and ACMs as toxic substances [9]. Later, in 2010, the Law on Toxic and Hazardous Substances began regulating asbestos and ACMs. The initial thrust of the government control of asbestos was a total banning of all types of asbestos in Mongolia. However, it failed due to inadequate preparation and lack of a national action plan on asbestos control. This paper explains the failure of such a total ban of asbestos in Mongolia.

In many countries the use of asbestos has been banned in industries such as power plant generation, construction and railways and appropriate substitutes have been found. As knowledge developed in Mongolia about the hazards of asbestos, and the political climate changed, effects to control asbestos became welcome.

## 2. Materials and Methods

All available literature on asbestos related studies conducted in Mongolia and legal regulations, national standards and guideline on asbestos control was reviewed. We searched available scientific literature published in international journals in Google Scholar and Pubmed using the key word, “asbestos Mongolia,” then selected five relevant articles for review [11,12,13,17,18]. Public health journals, research reports and other relevant literature published between 2010 and 2020 was reviewed in order to find scientific literature published locally. As a result, we found six articles related to asbestos [3,4,7,14,15,16,25,26,27,28]. We used the Mongolian Central System of Legal Acts^1^, in search of legal acts and regulations on asbestos and found one law [19], one administrative regulation [21] and three government resolutions [1,10,23] which are currently enforced to control asbestos. Information gathered from the review was reported as sections about asbestos consumption, high risk groups, asbestos related diseases and legislation on asbestos use.

## 3. Results

### 3.1 Asbestos consumption

Mongolia has widely used asbestos and ACMs for insulation at thermal power plants, piping systems, buildings and for construction materials since 196 [14]. Mongolia does not have an asbestos mine nor ACMs manufacturer [3,16]. All consumed asbestos and ACMs in the country are imported from other countries, mainly China and Russia which both mine, use and promote asbestos. The Mongolian Custom Authority started to register imports of asbestos and ACMs in 1996 [3,13]. Therefore, it is not possible to accurately estimate asbestos consumption before 1996. Damiran, et al reported that the average annual consumption was 423 tons between 1996 and 2004, then it dramatically increased to 7,709.5 tons between 2008 and 2009 before dropping to 1,748.8 until 2014 [13]. However, consumption could be underestimated due to under-registered imports and lack of registration of asbestos use. The US Geological Survey reported that the average annual asbestos consumption in Mongolia between 1998 and 2003 was 285.3 metric tons which is 1.5 times lower than Damiran reported in 2018 (420 tons) [13,18]. In 2011, Giang Ving estimated per capita asbestos consumption in Mongolia between 1994 and 2008 as 0.11 kg/capita/year, however, it is 4.5 times lower than Damiran reported (as 0.5 kg/capita/year) [13,18].

### 3.2 Occupational exposure groups

The main users of asbestos in Mongolia are the energy and construction sectors. It is also used in repair and maintenance shops for automobiles, trucks, heavy equipment, locomotives and trains using asbestos containing brake pads, piping, insulation, gaskets and other parts [3,4,13,15,16]. All employees of these companies are potentially exposed to airborne asbestos in workplaces whether directly handling asbestos or not. A study determined that the average exposure to airborne asbestos among pipeline insulators of thermal power plants was up to 0.93 f/cm^3^, which exceeds the eight-hour permissible Mongolian exposure limits of 0.1 f/cm^3^ [11]. Also, this study showed that asbestos concentrations were 0.39 to 1.48 f/cm^3^ at distances of 3, 4 and 20 meters from asbestos insulation work [11,15]. It showed other employees of thermal power plants who were not directly involved in asbestos handling work were exposed to asbestos at similar levels as insulation workers. Another research paper showed that the exposure level to airborne asbestos among workers at locomotive repair shops was found to be at 0.38 f/cm^3^. Construction workers were exposed to asbestos at 0.2 f/cm^3^ during removal work of asbestos containing board from building walls [7]. Also, the exposure levels among workers of construction material stores and at analytical laboratories were 0.04 and 0.01 f/cm^3^, respectively [7,15]. There is no known safe level for asbestos exposure. All these workers are at risk for ARDs such as lung cancer, mesothelioma and asbestosis. The National Asbestos Profile stated that over 14,800 workers of thermal power plants and heat distribution pipeline systems were exposed to asbestos at the workplace [3]. It is obvious that there are more workers exposed to asbestos and the number of people with community exposure is unknown.

### 3.3 Asbestos related diseases

At present Mongolia does not have registration and surveillance of well-known ARDs such as asbestosis, lung cancer and mesothelioma. Therefore, it is difficult to estimate nationwide prevalence of ARDs among the population. This paper reports cases of ARDs based on available data.

In 2010 the WHO conducted a study to determine asbestos related disease in the archive of the National Cancer Center between 1980 and 2010. As a result of the study, 29 confirmed cases of mesothelioma were identified among 21,973 patient records in the archive between 1994 and 2010 [29].

As a key indicator of asbestos related disease, 54 cases of mesothelioma were diagnosed at the National Cancer Center by pathological review of tissue samples between 1994 and 2013. The age specific incidence rate of mesothelioma was estimated at 19.2 per million per year which is 3.9 times higher than the world average estimated by the WHO [14].

A first internationally reported case of mesothelioma in Mongolia was published in 2015. A 47 year old woman was diagnosed with pleural mesothelioma after 27 years of exposure to only chrysotile asbestos at a thermal power plant [12].

It is difficult to estimate the total number of ARDs in Mongolia based on current limited data. However, the available literature clearly shows that cases of ARDs are already occurring in Mongolia and its incidence rate exceeded the world average.

### 3.4 Legal regulation of asbestos use

Before 1995, there was no law which regulated the use of toxic and hazardous chemicals including asbestos. The Mongolian Parliament approved the Law on Protection against Toxic Chemicals (LPTC) in 1995. This law was used to regulate issues related to toxic chemicals such as their production, use, trade, export and import [9]. There is no historical data that considered all types of asbestos as toxic chemicals since asbestos was not covered by this law.

In 2006, the LPTC was replaced by a new Law on Toxic and Hazardous Substances (LTHS). Under article 6.1.6 of the LTHS in 2007, the government approved lists of toxic and hazardous chemicals which are restricted and prohibited for use in Mongolia by Resolution No.95. However, these lists did not include any form of asbestos [1].

The Ministry of Environment is the main governmental body which is responsible for the implementation of this law [19]. In 2009, the Regulation on Export, Import, Transboundary Transportation, Production and Trade of Toxic and Hazardous Chemical was approved [21]. Under article of 2.1.1 of this regulation, The Ministry of Environment licenses companies for use, sale, manufacture or disposal of hazardous substances including chemicals restricted and prohibited to use. However, asbestos and ACMs were still not included in the scope of this regulation.

The Custom Authority has an obligation to register imported asbestos and ACMs as well as other goods imported into the country [22]. However, custom statistics on asbestos and ACMs imported only began and became available in 1994 [13].

In 2010, the Ministry of Environment proposed to add asbestos to the list of toxic and hazardous chemicals prohibited for use since it causes cancer. The government approved the proposed list with Resolution 192, and the use of asbestos was legally banned in 2010 [10]. But, there was no nationwide action plan on implementation of the government’s decision on banning asbestos and use of substitution materials in industrial sectors. After approval of Resolution 192, the government received pressure from industries regarding the banning of asbestos, especially from thermal power plants. The power plants complained that substitution materials are expensive and not compatible for use as thermal insulation on hot surfaces [13]. In addition, owners of thermal power plants and decision makers in Mongolia were not fully aware of the health hazards and economic burden from future use of asbestos. This economic pressure and lack of information remains a major difficulty in the total ban of asbestos in Mongolia. Due to this pressure from the energy sector, the government changed the legal status on asbestos in 2011 with Resolution 176 [23].

Article 3.1.4 of the LTHC states restrictions on chemicals for use as toxic and hazardous chemicals which are allowed to be used only at permitted places under strict control technology for approved purposes and in certain amount [19]. All forms of asbestos and ACMs are allowed for use only in thermal power plants, as thermal insulation, heat resistant materials and with licensed allowable amounts. Other industries are not allowed to use any form of asbestos. This is in spite of substitute materials being available. Also, no country truly has adequate controls in place, so workers continue to be exposed.

As a requirement of the LTHS and the Law on Occupational Safety and Hygiene, thermal power plants, which use asbestos, have to implement precautionary and protective measures including labelling, risk assessment, hazard communication, administrative and engineering controls, environmental monitoring, medical check-ups and training. The Governmental Agency on Specialized Inspection monitors the use of restricted toxic and hazardous chemicals, including asbestos, and enforces the legal regulations [20].

In 2015 asbestos was added to the Occupational Hygiene Standards MNS 4990 which regulates the occupational exposure limits for hazardous substances [25]. Furthermore, guideline standards of analytical and sampling methods for asbestos measurement were translated into Mongolia and were adopted as national standards [26,27].

The most vulnerable groups to asbestos hazards are workers at asbestos using workplaces. In 2017, the Mongolian National Association of Occupational Hygienists developed a Guideline of Safety Procedure for Asbestos Handling Work to protect workers who are involved in asbestos related jobs. The National Committee of Occupational Safety and Hygiene led by the Vice Minister of Labor and Social Security approved this guideline as a recommendation under Resolution No.01 in 2020 [28]. The Guideline includes essential precautionary measures of occupational hygiene such as exposure assessment, enclosure or isolation of asbestos involved work, training of workers, personal protective equipment, safe work practices, negative pressure units, removal of ACMs and handling of asbestos containing wastes. Table 1 summarizes regulations on asbestos control in Mongolia.

**Table 1:** Legal regulations, standards and guideline utilized in asbestos control in Mongolia.


APPROVED YEAR	LEGAL REGULATION, STANDARD, GUIDELINE	STATUS OF IMPLEMENTATION

2006	Law on Toxic and Hazardous Substances approved by Mongolian Parliament and latest amendment was in 2020 [19].	Mandatory

2007	List of Toxic and Hazardous Chemicals Restricted to use in Mongolia, Annex 2 of Resolution No. 95 of Mongolian Government and amended in 2011 [1]	Mandatory

2008	Law on Occupational Safety and Hygiene approved by Mongolian Parliament and amended in 2018 [20]	Mandatory

2009	Regulation on Export, Import, Transboundary Transportation, Production and Trade of Toxic and Hazardous Chemicals approved by Order No. 334/104 of Ministers of Environment and Tourist, and Foreign Affairs and amended 2019 [24]	Mandatory

2015	MNS 4990: 2015. Occupational Safety and Hygiene Standard (Occupational exposure limit for air borne asbestos) [25]	Mandatory

2015	MNS ISO 8672:2015. Air quality – Determination of the number concentration of airborne inorganic fibres by phase contrast optical microscopy – Membrane filter method [26].	Voluntary

2015	MNS ISO 10312:2015. Ambient air-Determination of asbestos fibres – Direct transfer transmission electron microscopy method [27]	Voluntary

2020	Safety Procedure for Asbestos Handling Work to recommended by The National Committee of Occupational Safety and Hygiene in 2020 [28]	Voluntary


## 4. Discussion

The total banning of asbestos was not successful in Mongolia and is still legally allowed for some use. However, the policy on asbestos control influenced a dramatic decrease in asbestos consumption. A recently published research article showed that annual asbestos consumption in 2014 has declined by four times compared to peak use in 2008 [13].

Initiatives were started to protect workers and the general community from exposure to asbestos, and to monitor exposure, register asbestos related diseases and enhance national capacity to lead with asbestos hazards. Since approval of the first regulation on asbestos control (2010), training and workshops were organized to enhance the national capacity on asbestos control, measurements and diagnose and register ARDs with support from WHO and other institutions, including Japanese and US universities [3,4,25,26,27,28,29]. Awareness raising activities on asbestos hazards were organized to educate employers, main asbestos users, workers and the public.

The construction sector was not allowed to continue the use of asbestos. As thermal insulation and heat resistant materials, home use of asbestos containing materials is no longer in common use [1,23]. As reported by the Governmental Agency for Specialized Inspection, thermal power plants have started to substitute materials and reduce consumption of asbestos and ACMs.

Mongolia still needs an integrated policy or regulations regarding asbestos control including effective control of asbestos exposure, health surveillance of asbestos related diseases, compensation and exposure monitoring and prevention of economic loss. It needs a policy and facility to properly dispose of asbestos containing materials that are removed. The role of governmental instructions and regulations are needed to control asbestos activities, and it may need to be defined in legal regulations.

## 5. Conclusions

Prior to the banning of asbestos in Mongolia in 2010, proper preparations for the effects on the industry were not considered. Having policies not supported by practical measures in developing countries such as Mongolia, are by themselves, insufficient to protect workers and others. Therefore, main asbestos users such as power plants were not prepared to replace thermal insulation materials and were not aware of potential future health and economic consequences. This situation caused a step backward on asbestos control and allowed the use of asbestos again in 2011. Also, the legal situation in Mongolia does not put the same pressures on Mongolian users of asbestos as has occurred in developed countries.

## Footnotes

www.legalinfo.mn.
